# Meningeal immunity and neurological diseases: new approaches, new insights

**DOI:** 10.1186/s12974-023-02803-z

**Published:** 2023-05-25

**Authors:** Yun Su, Huimin Zheng, Changhe Shi, Xinwei Li, Shuyu Zhang, Guangyu Guo, Wenkai Yu, Shuo Zhang, Zhengwei Hu, Jing Yang, Zongping Xia, Chengyuan Mao, Yuming Xu

**Affiliations:** 1 Department of Neurology, The First Affiliated Hospital of Zhengzhou University, Zhengzhou University, No.1 Eastern Jian-She Road, Zhengzhou, 450052 Henan China; 2 The Academy of Medical Sciences of Zhengzhou University, Zhengzhou University, Zhengzhou, 450052 Henan China; 3 Henan Key Laboratory of Cerebrovascular Diseases, The First Affiliated Hospital of Zhengzhou University, Zhengzhou University, Zhengzhou, 450052 Henan China; 4 Neuro-Intensive Care Unit, The First Affiliated Hospital of Zhengzhou University, Zhengzhou University, Zhengzhou, 450052 Henan China; 5 Sino-British Research Centre for Molecular Oncology, National Centre for International Research in Cell and Gene Therapy, School of Basic Medical Sciences, Academy of Medical Sciences, Zhengzhou University, Zhengzhou, 450052 Henan China; 6 NHC Key Laboratory of Prevention and Treatment of Cerebrovascular Diseases, Zhengzhou, 450052 Henan China

**Keywords:** Meninges, Meningeal immunity, Single-cell technologies, Neurodegenerative disease, Neuroinflammatory disease

## Abstract

The meninges, membranes surrounding the central nervous system (CNS) boundary, harbor a diverse array of immunocompetent immune cells, and therefore, serve as an immunologically active site. Meningeal immunity has emerged as a key factor in modulating proper brain function and social behavior, performing constant immune surveillance of the CNS, and participating in several neurological diseases. However, it remains to be determined how meningeal immunity contributes to CNS physiology and pathophysiology. With the advances in single-cell omics, new approaches, such as single-cell technologies, unveiled the details of cellular and molecular mechanisms underlying meningeal immunity in CNS homeostasis and dysfunction. These new findings contradict some previous dogmas and shed new light on new possible therapeutic targets. In this review, we focus on the complicated multi-components, powerful meningeal immunosurveillance capability, and its crucial involvement in physiological and neuropathological conditions, as recently revealed by single-cell technologies.

## Introduction

The meninges comprise a triple layer of membranes that jointly envelop the central nervous system (CNS) [1]. Previously, they have been regarded as a supportive barrier protecting the CNS from potential threats and segregating it from the periphery. Until recently, accumulating evidence has indicated that the meninges are also equipped with a rich repertoire of immune cell populations and functional lymphatic networks [2, 3], thus actively enabling meningeal immunity [4]. Since meningeal immunity has attracted considerable attention and become a research focus, this immunologically active barrier has been growingly recognized to be involved in modulating proper brain functions and social behavior [5], performing constant immune surveillance of the CNS [6, 7], and participating in several neurological diseases [5–7]. Therefore, meningeal immunity is increasingly assumed to play a key role in both beneficial and detrimental mechanisms in the context of homeostasis and inflammation. However, the precise cellular and molecular mechanisms underlying the meningeal immunity effect on the CNS under physiological and pathological conditions, remain poorly understood, due to their complexity and the limitations of the previous prevailing methodology performed on the whole tissue.

Recently, single-cell omics and other advances in technology have been developed. In particular, single-cell technologies such as single-cell RNA sequencing (scRNA-seq), single-cell B cell receptor sequencing (scBCR-seq), and cytometry by time-of-flight mass spectrometry (CyTOF) have enabled researchers to overcome these limitations. Specifically, scRNA-seq allows quantifying the transcriptional profiles at the single-cell resolution, recognizing unappreciated cell clusters with high-dimensional throughput, and characterizing cellular heterogeneity even within apparently homogeneous populations. Moreover, it allows the computational analysis of potential intercellular interactions [8, 9]. In addition, scBCR-seq shares a similar experimental strategy and can reveal the B-cell receptor repertoire diversity and their antigen specificity [10]. Furthermore, CyTOF allows a simultaneous assessment of the proteomic patterns of more than 40 markers expressed in thousands of cells [11, 12]. These techniques have remarkably advanced our understanding of the cellular and molecular landscapes of complex immune compartments, such as the meninges, thanks to the single-cell resolution and high-throughput data.

Here, we summarize recent studies using the state-of-the-art single-cell technologies on the meningeal environment (Table 1), and provide new insights into the complex multi-components, powerful immunosurveillance capability, and crucial involvement of the meningeal immunity under physiological and neuropathological conditions.

**Table 1:** Overview of recent studies with single-cell analysis of meningeal immunity

Single-cell technology	Tissue origin	Single-cell isolation	Protocol	References
scRNA-seq	Leptomeninges from WT and EAE mice models	FACS sorting of CD45^+^ immune cells	CEL-Seq2	2019 [37]
	Dural and subdural meninges from WT C57BL/6 mice	FACS sorting of CD45^+^ immune cells	10 × Chromium	2019 [18]
	Dural meninges from young and old WT C57BL/6 mice	FACS sorting of CD45^+^CD3e^+^TCRβ^+^ T cells	10 × Chromium	2021 [26]
	Dural meninges from young and old WT C57BL/6 mice	FACS sorting of CD45^−^CD31^−^CD13^+^ mural and CD45^−^CD31^+^ endothelial cells	10 × Chromium	
	Whole dural meninges from young and old WT C57BL/6 mice	FACS sorting of DAPI^−^ cells	10 × Chromium	
	Dural sinuses from WT C57BL/6 mice	FACS sorting of CD45^+^CD11b^+^Ly6G^−^ myeloid cells	10 × Chromium	
	Whole dural meninges from WT C57BL/6 mice	FACS sorting of DAPI^−^ cells	10 × Chromium	2021 [14]
	Spinal cord tissue from EAE parabionts	FACS sorting of DAPI^−^CD45^hi^IV-CD45^−^GFP^−^ and DAPI^−^CD45^hi^IV-CD45^−^GFP^+^ singlets	10 × Chromium	
	Spinal cord tissue from WT parabionts after spinal cord injury	FACS sorting of DAPI^−^CD45^hi^IV-CD45^−^GFP^−^ and DAPI^−^CD45^hi^IV-CD45^−^GFP^+^ singlets	10 × Chromium	
	Dural meninges from WT C57BL/6 mice	Unsorted	10 × Chromium	2021 [15]
	Dural meninges and subdural meninges from WT C57BL/6 mice	FACS sorting of CD45^+^CD45iv^−^ tissue-resident leukocytes	10 × Chromium	2021 [24]
	Six scRNA-seq data set integration	FACS sorting	10 × Chromium; MARS-seq	2022 [47]
**scBCR-seq**	Dural meninges from young and old C57BL/6 mice	Unsorted	10 × Chromium	2021 [15]
	Dural meninges from unimmunized and EAE mice models	Unsorted	10 × Chromium	2021 [24]
**CyTOF**	Mixed brain, enveloping meninges and choroid plexus from WT C57BL/6 mice	Unsorted	CyTOF1 mass cytometer	2017 [19]
	Mixed brain and enveloping meninges from WT, geriatric, and EAE mice models	Unsorted	Third-generation Helios mass cytometer	2018 [20]
	Mixed CNS parenchyma and enveloping meninges from WT, EAE, and HD mice models	Unsorted	CyTOF2 mass cytometer	2018 [21]
	Mixed brain, enveloping meninges and choroid plexus from WT C57BL/6 mice	Unsorted	CyTOF1 mass cytometer	2018 [22]
	Dura mater from WT C57BL/6 mice	Unsorted	CyTOF2 mass cytometer	2021 [15]

*scRNA-seq* single-cell RNA sequencing; *WT* wild-type; *EAE* experimental autoimmune encephalomyelitis; *FACS* Fluorescence-activated cell sorting (FACS); *CD* cluster of differentiation; *CEL-Seq2* cell expression by linear amplification and sequencing version 2; *TCR* T cell receptor; *DAPI* 4′,6-diamidino-2-phenylindole; *hi* highly expressed; *iv* intravenously injected; *MARS-seq* massively parallel single-cell RNA sequencing; *CyTOF1* cytometry by time-of-flight mass spectrometry generation 1; *CyTOF2* cytometry by time-of-flight mass spectrometry generation 2; *HD* Huntington disease

## Anatomic and cytological components of the meningeal immunity

### Meningeal structural composition

**Fig. 1:**
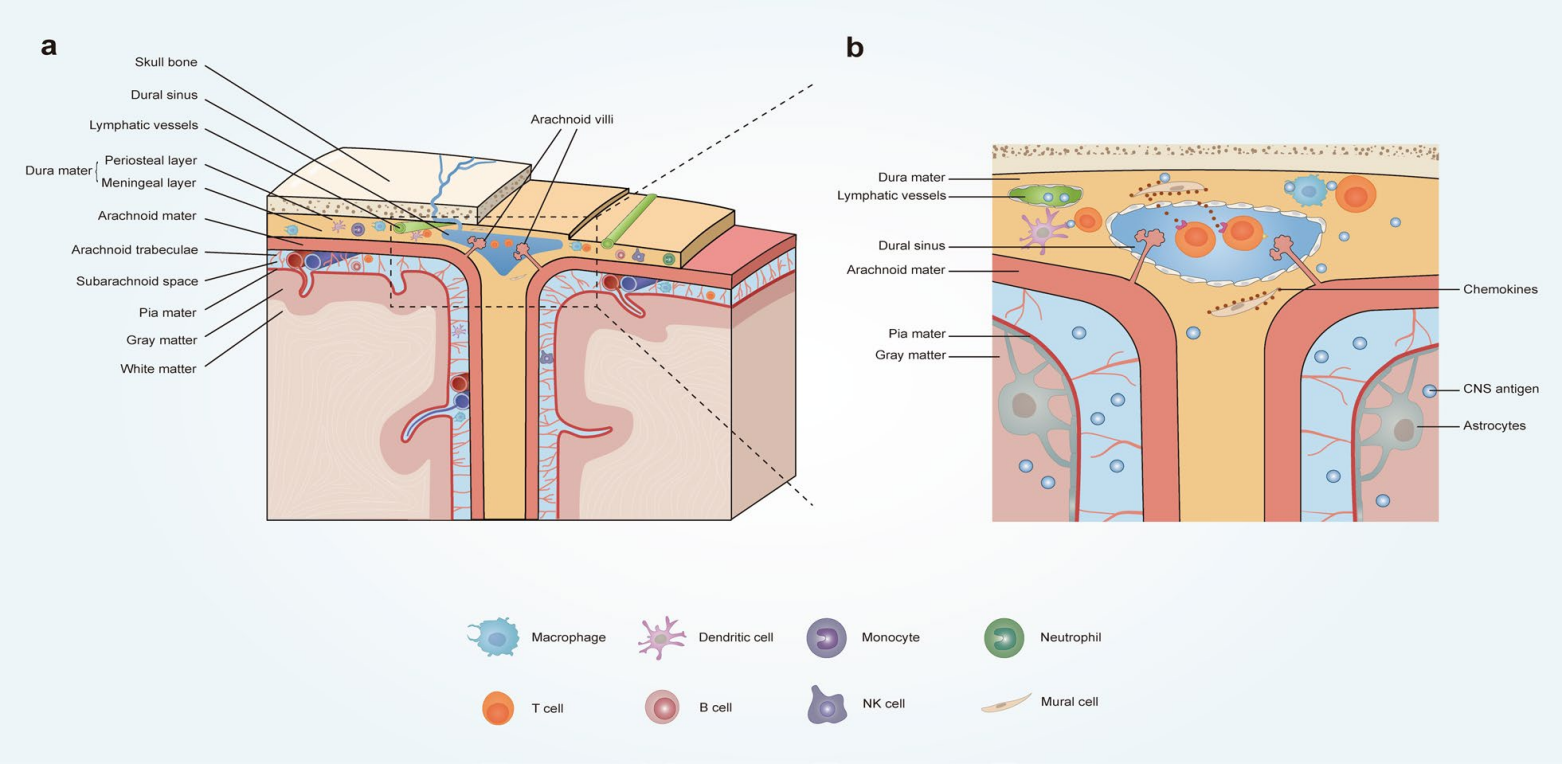
Structure and function of meninges in homeostatic brain. **a** Meningeal structure and immune composition from perspective of the coronal section of human’s skull. The dura mater residing underneath the skull bone consists two layers: the periosteal and meningeal layer. Dural sinuses reside in specific sites, where these two layers separate from each other, and drain cerebral venous blood towards the systemic circulation. Meningeal lymphatic vessels run along the dural sinuses. The arachnoid mater is the middle layer of meninges, which effectively separates the dura from the subarachnoid space filled with the cerebrospinal fluid (CSF). The arachnoid mater penetrates deep into the dural sinuses and forms numerous arachnoid villi though which part of CSF flows into the dural sinuses and further into the systemic circulation. The pia mater intimately associated with the brain and spinal cord is the deepest layer of meninges. In addition, diverse immune cells are present in the distinct meningeal layers in homeostasis. The schemes are based on evidence in experimental animal models. **b** Partial enlarged perspective of the coronal section centered on dural sinuses. Dural sinuses are immune hubs in which immune surveillance of homeostatic CNS works. Circulating T cells are recruited to the dura by chemokines released from dural stromal cells, such as mural cells, and accumulate around the dural sinuses in homeostasis. Both dural lymphatic vessels and arachnoid villi are involved in CSF circulation, which may allow the drainage of CNS-derived antigens in the CSF to perisinusal dura. Sinus-associated antigen-presenting cells including macrophages and dendritic cells capture CNS-derived antigens and present these antigens to perisinusal T cells, thus allowing meningeal immune surveillance of homeostatic CNS

The meninges are composed of three structurally distinct layers: the dura, arachnoid and pia mater [1] (Fig. 1a). These membranes, and particularly the dura mater, harbor a diverse array of both innate and adaptive immune cells, such as myeloid and lymphoid populations. Furthermore, this outermost meningeal layer includes fenestrated vasculature and functional lymphatic vessels [3], and harbors adjacent bone-marrow-derived immune cells at the skull-dura interface [13–15].

The middle meningeal layer is the arachnoid mater, primarily lining the subarachnoid space through which the cerebrospinal fluid (CSF) circulates. The meningeal lymphatic vessels (MLVs) provide a newly discovered route for the uptake and drainage of cerebral CSF, immune cells, and CNS-derived antigens into the cervical lymph nodes (cLNs), particularly the deep cervical lymph nodes (dcLNs) [3, 16].

Finally, the innermost layer is the pia mater, adhering closely to the CNS parenchyma. Together, the arachnoid and pia mater are known as leptomeninges or subdural meninges. When preparing single-cell suspensions for the experimental sequencing procedures, it is feasible to remove the dura mater from the skull, and scrape thin slices from the dorsal cortex for enriched subdural meninges in mice [17].

### Characteristics of the meningeal immune cells identified with single-cell techniques

Recent studies have provided detailed information on the meningeal immune cell types and their specific characteristics at a single-cell level (Table 2). A recent study provided deep insights into the specific immune landscape of homeostatic boundary areas using an elegant combination of these micro-dissected regions from 9-week-old mice of wild-type C57BL/6J, flow-cytometry sorting for CD45+ immune cells, and scRNA-seq. These border regions included the dura mater (D), subdural meninges (SDM), and choroid plexus (CP) [18]. The dura mater was enriched with numerous border-associated macrophages (D-BAMs), dendritic cells (DCs), monocytes, neutrophils and lymphocytes, subdivided into multiple subtypes based on specific gene signatures [18]. The dural macrophages were subdivided into two subtypes based on their low or high major histocompatibility complex (MHC) class II expression (D^lo^-BAMs and D^hi^-BAMs, respectively), suggesting the potential for antigen presentation by the latter [18].

**Table 2:** Characteristics of meningeal immune cells under homeostasis

	Cell clusters (percentage)	Cell ontogeny	Transmigration pathway	Cell subsets	Subset/tissue-specific signatures	References
Dura mater	BAMs (35%)	Bone marrow	Peripheral circulation	D^hi^-BAM	*H2-Aa, Cd74, Ccr2, H2-Eb1*	2019 [18]
				D^lo^-BAM	*Ccl8, Pla2g2d, Cfp*	
	DCs (19%)	–	–	cDC1	*Flt3, Xcr1*	
				cDC2	*Flt3, Cd209a, Itgax*	
				migDCs	*Ccr7, Nudt17*	
				pDCs	*Siglech, Ccr9, Pacsin1*	
	Monocytes (16%)	Adjacent skull and vertebral bone marrow^#^; peripheral blood*	Bone marrow-dura channels^#^; peripheral circulation*	Classical monocytes	*Ly6c2*	2021 [14], 2019 [18]
				Non-classical monocytes	*Itgal*	
				Intermediate monocytes	*Fcgr1*	
	Neutrophils (12%)	Adjacent skull and vertebral bone marrow^#^; peripheral blood*	Bone marrow-dura channels^#^; peripheral circulation*	Neutrophils	*Ly6g, Itgam*	
	B cells (7%)	Adjacent skull and vertebral bone marrow^#^; peripheral blood*	Bone marrow-dura channels^#^; peripheral circulation*	Pro-B cells	*Vpreb1, Igll1, Dntt, Lef1, Tspan13, Smarca4*	2021 [15], 2019 [18]
				Pre-B cells	*Dnajc7, Rag1, Cecr2, Sox4, Myb*	
				Immature B cells	*Ms4a1, Ly6d, Hck, Cd24a, Spib, Ccnd2, Cd72*	
				Mature naïve B cells	*Rps29, Rpl38, Rps27, Rpl21, Igkc*	
				Mitotic B cells	*Top2a, Mki67*	
	T cells (4%)	Peripheral blood	Peripheral circulation	T cells	*Cd3e*	2019 [18]
	NK cells (4%)	–	–	NK cells	*Klrb1c*	
Subdural meninges	Microglia (67%)	Embryo	Tissue residency	Microglia	*Sparc, Mertk, Cx3cr1, Csf1r, Itgam, Aif1, Cd68, Fcgr1*	2019 [18]
	BAMs (23%)	Embryo	Tissue residency	BAMs	*Lyve1, P2rx7, Egfl7*	
	DCs (2.6%)	–	–	cDC1	*Flt3, Xcr1*	
				cDC2	*Flt3, Cd209a, Itgax*	
	Monocytes (2%)	–	–	Classical monocytes	*Ly6c2*	
				Non-classical monocytes	*Itgal*	
	T cells (3%)	–	–	T cells	*Cd3e*	
	B cells (1%)	–	–	B cells	*Cd19*	
	NK cells (1%)	–	–	NK cells	*Klrb1c*	

*scRNA-seq* single-cell RNA sequencing; *BAMs* border-associated macrophages; *D*^*hi*^*-BAM* dural macrophages with high expression of major histocompatibility complex class II; *D*^*lo*^*-BAM* dural macrophages with low expression of major histocompatibility complex class II; *DCs* dendritic cells; *cDC1* conventional dendritic cell subset 1; *cDC2* conventional dendritic cell subset 2; *migDCs* migratory dendritic cells; *pDC* plasmacytoid dendritic cells; *NK cells* natural killer cells

^#^Immune cells from adjacent skull and vertebral bone marrow transmigrate to the dura through bone marrow-dura channels

*Immune cells from peripheral blood transmigrate to the dura through peripheral circulation

Bulk RNA sequencing has revealed hundreds of differentially expressed genes (DEGs) between D^lo^-BAMs and D^hi^-BAMs; these findings complemented the scRNA-seq results and demonstrated intra-border heterogeneity [18]. The BAMs exhibited tissue-specific transcriptional profiles (Table 2); however, scRNA-seq revealed a core BAM genetic signature including *Apoe*, *Ms4a7*, *Ms4a6c*, *Lyz2* and *Tgfbi*, regardless of tissue localization. DCs and monocytes were also prevalent in the dura mater, whereas T and B lymphocytes are relatively rare [18]. Despite the extensive presence of microglia in enriched SDMs, overcoming other immune populations and indicative of cortical contamination, diverse immune cell types were detected in the SDMs based on key marker genes (Table 2). In contrast, CyTOF failed to offer a specific perspective on the meningeal immunity environment, because the brain parenchyma and enveloping meninges from experimental mice were collected simultaneously in one sample [19–22]. Anatomically defined and precise dissection, combined with CyTOF, may yield specific insights into the immune landscape of the CNS parenchyma and its associated boundaries at the protein level.

Potential cell sources in the meningeal immune compartment are also a main area of concern. Using reporter mouse models, subdural (SD) -BAMs were found to be embryonically derived, whereas D-BAMs underwent gradual replacement via bone marrow-derived precursors with subtype-distinct kinetics [18]; this finding was confirmed by another study using single-cell mass cytometry [20]. Moreover, recent studies have provided a first glimpse into dural monocytes, neutrophils and B cells originating from adjacent skull and vertebral bone marrow niches, via direct dura-bone marrow connections, in physiological conditions and CNS dysfunction [14, 15]. However, the same scenario was not observed for T lymphocytes, suggesting a peripheral blood origin. Previously, confocal microscopy and in vivo imaging had revealed the presence of ossified vascular channels traversing the inner skull cortex and further connecting the marrow cavities with the dura mater in mice [13, 23]. Similar channels were also found in human craniectomy specimens, with diameters five times larger than those seen in mice [13]. This novel finding suggests the hypothesis that these concealed channels may serve as shortcuts for the migration of bone marrow-derived cells towards the CNS boundaries under physiological and pathological conditions. Moreover, two other studies observed similar vascular channels directly connecting the skull and vertebral bone marrow to the adjacent dura, and a bone marrow-derived dural myeloid reservoir (monocytes and neutrophils) and lymphoid continuum (B cells). Furthermore, these adjacent bone marrow-derived immune cells could be mobilized to penetrate the CNS parenchyma, supplementing their peripheral counterparts during CNS dysfunction [14, 15]. Another study using scRNA-seq of tissue-resident leukocytes, extracted from the CNS parenchyma of wild-type rats and its borders, revealed that the dura mater typically contains a large population of B cells and B-lineage progenitors at the pro-B cell stage [24], confirming the finding that multiple developmental stages of B cells populate the dura [15]. The consistency observed among various studies suggests the robustness and reliability of the scRNA-seq analysis. In addition, scRNA-seq analysis identified dural signals crucial for local recruitment and development, including the CCL2-, CCL12-, and CCL8-CCR2 axes assigned to monocytes, CCL6-CCR1 to neutrophils, and CXCL12-CXCR4 to B cells [14, 15], opening promising avenues to manipulate the meningeal immunity.

## scRNA-seq suggesting dural sinuses as centers for immune surveillance

It is tempting to speculate that the dura mater, leptomeninges, and CP may serve as unique interfaces, where the immune surveillance is active [4]. However, the tight junction within the leptomeningeal vascular endothelium and the epithelium lining the CP [25], make the respective structures less likely to support extensive T-cell trafficking, leaving the dura mater as the most suitable candidate.

The dural sinuses have recently been identified as immune hubs, where circulating T cells constantly access CNS-enriched antigens with the help of local antigen-presenting cells (APCs), allowing homeostatic immune surveillance [26] (Fig. 1b). More specifically, scRNA-seq combined with ligand-receptor inference analysis revealed both physical and signaling interactions between the dural stromal cells and immune populations in young mice. In particular, the CXCL12-CXCR4 signaling contributes greatly to the recruitment of circulating T cells into the dura [26]. The dural stromal niche contains endothelial populations and mural subtypes that closely regulate the homeostatic tissue immunity. After adhesion and arrest mediated by high adhesion molecule expression on the dural sinus endothelium, T cells extravasated through the dural sinuses, accumulated in proximity to the dural sinuses, and were steadily replenished from the periphery [26]. CNS-derived antigens in the CSF were also drained into the perisinusal dura and captured by sinus-associated APCs. The latter were identified as macrophages and DCs because of their high expression of MHC class II, as shown by scRNA-seq analysis [26], in line with the abovementioned study [18]. The T cells interact with the APCs and recognize cognate antigens, displaying tissue-resident phenotypes and effector functions, thus enabling efficient immune surveillance of the CNS in homeostasis [26]. The perisinusal localization of APCs and similar accumulation of brain-enriched proteins were also observed within the dura mater in human postmortem samples [26], suggesting the presence of shared mechanisms underlying the immune surveillance at the dural sinuses. In addition, the efflux of brain-enriched antigens from the CSF to the perisinusal dura precedes the lymphatic drainage [26], suggesting that CNS-derived antigens are monitored at the CNS boundaries prior to the peripheral immune system. These mechanisms suggest a crucial role of the meninges, as they provide immune responses before the peripheral immune system. In the event of aging or neuroinflammatory conditions, this neuroimmune interface can adapt promptly.

Therefore, recent studies using comprehensive scRNA-seq analysis attributed the CNS immune surveillance to dural sinuses and explained the cellular and molecular cues involved in these concentrated dural immune hubs. Other molecules critical for immune-cell multi-step trafficking have been reviewed elsewhere [4, 27].

## Meningeal immunity participating in neurological diseases

**Fig. 2:**
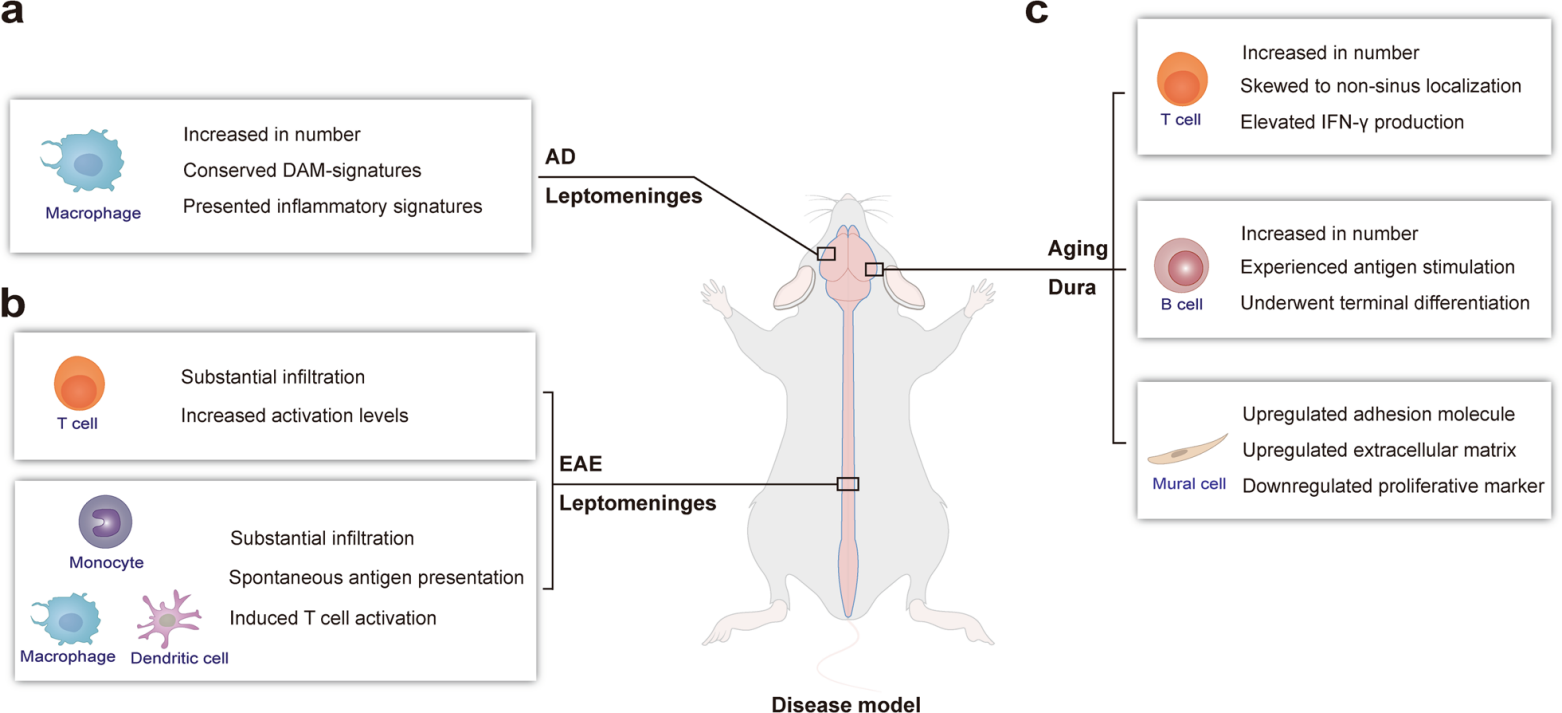
Alterations of meningeal immunity components in neurological diseases via single-cell analysis. **a** Involvement of disease-inflammatory macrophages in the leptomeningeal inflammatory processes in mouse models of Alzheimer’s disease (AD) expressing five human familial AD gene mutations. **b** Involvement of T cells and myeloid cells in the leptomeningeal inflammatory processes in active and transfer mouse models of experimental autoimmune encephalomyelitis (EAE). **c** The cellular and molecular changes of dural immune and stromal cells during aging

Recent advances in meningeal immunity research have been expedited by novel single-cell technologies, particularly regarding neuroinflammatory or neurodegenerative conditions and aging. Therefore, we will review the previously prevailing viewpoints in brief, and then thoroughly delineate the new insights into the involvement of meningeal immunity in widely studied diseases, such as multiple sclerosis and experimental autoimmune encephalomyelitis, aging, and Alzheimer’s disease (Table 3, Fig. 2).

**Table 3:** Involvement of meningeal immunity in neurological diseases via single-cell analysis

Disease model	Cell type	Evolving insights of meningeal immunity	References
MS and EAE	Immune cells	Myeloid populations within the leptomeninges displayed disease- and tissue-specific transcriptional kinetics in EAE mice	2019 [37]
		Adjacent bone marrow-derived myeloid cells residing in the dura under homeostasis further infiltrated the CNS parenchyma and exhibited regulatory phenotypes in EAE mice	2021 [14]
		Leptomeningeal inflammation was far more severe than dural counterparts in both EAE mice and MS patients	2022 [38]
Aging	Immune cells	Dural T cells increased in number, skewed towards non-sinus regions, and enhanced IFN-γ expression in old mice	2021 [26]
		Age-associated B cells derived from the blood accumulated in the dura, showed antigen-experienced pattern and underwent differentiation into plasma cells in old mice	2021 [15]
		Disease inflammatory macrophages highly expressing inflammation-related genes increased in number during aging	2022 [47]
	Nonimmune cells	Dural stromal populations upregulated adhesion molecules and extracellular matrix at non-sinus sites in old mice	2021 [26]
AD	Immune cells	Impaired meningeal lymphatics induced aberrant activation of microglia in 5 × FAD mice	2021 [45]
		Disease inflammatory macrophages highly expressing inflammation-related genes were present in the leptomeninges of both 5 × FAD mice and AD patients	2022 [47]

*MS* multiple sclerosis; *EAE* experimental autoimmune encephalomyelitis; *CNS* central nervous system; *IFN-γ* interferon-γ; *AD* Alzheimer’s disease; *5 × FAD* five human familial AD gene mutations

### Multiple sclerosis (MS) and experimental autoimmune encephalomyelitis (EAE)

MS is a chronic autoimmune CNS disorder caused by autoreactive lymphocytes [28]. The phenotypic classifications of MS include clinically isolated syndrome, relapsing–remitting multiple sclerosis, primary-progressive multiple sclerosis (PPMS), secondary progressive multiple sclerosis (SPMS) and radiologically isolated syndrome [28]. Its neuropathological hallmarks include inflammation, demyelination, neuronal and axonal loss and astrocytic gliosis [28]. In addition, EAE is a well-known experimental model of MS that can be actively or passively induced [29].

A new field of research was opened by the first immunohistochemical evidence that ectopic lymphoid follicle-like structures are present in the cerebral leptomeninges of patients with SPMS [30]. Accumulating studies have indicated that these ectopic B-cell follicles are strongly associated with a more aggressive clinical course, including younger age of onset, more severe disability states, and death. The cerebral cortical pathology, including gray matter demyelination, microglial activation, and neuronal loss, is also more severe [31–33]. Meningeal immunity may contribute to the pathogenesis of cortical pathology through immune cell-derived cytokines mediating cytotoxicity. This hypothesis is supported by the presence of meningeal inflammation preceding the parenchymal immune infiltrations, its topographical proximity to cortical lesions (predominantly subpial gray matter demyelination), and neuronal loss and microglial activation in a gradient [31, 32]. A similar scenario of diffuse meningeal inflammation was observed in patients with PPMS, even in the early stages [34, 35], highlighting the extensive involvement of meningeal immunity. In addition, magnetic resonance imaging has provided a non-invasive methodology for the in vivo visualization of leptomeningeal inflammation [36]. However, these studies are insufficient for the comprehensive characterization of cellular composition, organization, function, and coordination in the entire meningeal environment under autoinflammatory conditions.

Another factor to investigate is the possible involvement of the bone marrow-derived myeloid cells within the dura in the neuropathological processes. Vertebral bone marrow-derived monocytes were observed to reach the CNS borders via direct bone marrow-dura channels and then infiltrate the inflamed spinal cord in EAE mice [14]. Analysis with scRNA-seq of spinal cord-infiltrating immune cells in these mice revealed that monocytes from both blood and bone marrow were the most common cell types and presented distinct phenotypes and functions [14]. In particular, DEG analysis demonstrated increased levels of myeloid and lymphocytic chemokines and proinflammatory cytokines in blood-derived monocytes, promoting leukocyte migration and adhesion, further cytokine production, and T-cell activation [14]. This finding suggests that blood-derived monocytes may play a proinflammatory role in EAE. In contrast, the bone marrow-derived monocytes showed phenotypes aimed at regulatory functions rather than inflammation. The same characteristics were observed in models of spinal cord injury [14]. Therefore, the dural myeloid cells derived from adjacent bone marrow niches may serve as an emergency reserve, triggered by injury and neuroinflammation.

A recent scRNA-seq study of several CNS compartments isolated from EAE mice comprehensively characterized the transcriptional profiles and dynamics of myeloid populations during neuroinflammation [37]. Ten cell subtypes were identified in the leptomeninges in consecutive stages of the disease, including macrophages with homeostatic and disease-associated phenotypes [37]. The latter presented a significant induction of inflammatory chemokines and MHC class II compared to their homeostatic counterparts, suggesting disease stage-related alterations and proinflammatory reactivity [37]. These tissue-resident macrophages in the leptomeninges showed local proliferation and considerable self-maintenance during EAE, indicating an embryonic origin, as previously described [18]. Several types of monocytes, including Ly6C^hi^, monocyte-derived macrophages, and dendritic cells, were identified based on their additional expression of macrophage- or dendritic cell-derived genes (such as *Mertk* and *Cd209a*, respectively), implying a local ongoing differentiation [37]. DCs were undetectable in the homeostatic leptomeninges; however, their density dramatically increased during EAE. All macrophages, monocytes, and monocyte-derived cells exhibited high expression of antigen-presentation signatures, though the presentation to T cells was ultimately attributed to hematopoietic stem cell-derived circulating myeloid cells rather than to tissue-resident macrophages [37]. In addition, circulating myeloid cells were first observed in proximity to the leptomeninges [37], suggesting that these structures may be an entry point for the peripheral immune system. Collectively, these results have improved our understanding of the cellular components, their context-dependent transcriptional alterations, and potential functions and interactions in the meningeal scene during neuroinflammation.

A strikingly different contribution of each meningeal layer to CNS autoimmunity has recently been identified [38]. A substantial infiltration of myeloid and T cells has been observed in the spinal cord leptomeninges and parenchyma in active and transfer EAE models, irrespective of antigen specificity, whereas the dura presented sparse inflammation [38]. A similar scenario was observed in patients with chronic MS [38]. The infiltration of antigen-specific T cells was less prominent in the dura than in the leptomeninges in all stages of EAE; the activation level of effector T cells was also lower in the dura, regardless of the induced models or species [38].

Despite the higher permeability of the dural vasculature, the interactions between pathogenic T cells and vascular endothelium were weaker in the dura than in the leptomeninges. This finding may be partly explained by the lower expression of tight junction molecules and firm adhesion factors in the dural vasculature, as shown by transcriptome analysis [38]. The same analysis also excluded the possibility of inherent functional deficits in both APCs and T cells. A reactive T-cell stimulation test in vitro indicated that dural APCs could present antigens, though they required additional autoantigen administration to evoke the stimulatory potential, whereas the leptomeningeal APCs exhibited full competence and spontaneously induced T-cell activation [38]. This phenomenon was further confirmed in vivo and helped attribute the reduced interactions between effector T cells and APCs to insufficient amounts of autoantigens in the dural APCs [38].

These findings (different degree of inflammatory infiltration, activation level of effector T cells, and antigen presentation capacity of APCs between the dura and leptomeninges) were also observed in chronic EAE models [38]. Therefore, the uneven involvement of the different layers compellingly reshapes our concept of the meningeal role in CNS autoimmunity.

### Aging

Aging is a natural and inevitable process characterized by a decline in the immune system function and chronic low-grade inflammation [39].

Age-related meningeal lymphatic dysfunction has been demonstrated in elderly mice [40, 41]. Specifically, these mice exhibited a decreased diameter and coverage of the MLVs with consequent impaired CSF drainage into the dcLNs [41], and when the MLV function was damaged, the mice exhibited cognitive deficits. RNA-seq analysis revealed that the signaling pathways were downregulated by examining the lymphangiogenic growth factors in lymphatic endothelial cells (LECs) from aged mice [41]. Furthermore, treatment with vascular endothelial growth factor C (VEGF-C), a molecule prompting lymphatic vessel growth, reversed the structural changes observed, increased the lymphatic drainage, and improved the cognitive performance [41]. Therefore, the meningeal lymphatic drainage network during aging becomes insufficient to maintain adequate fluid and protein homeostasis, and its alteration is related to cognitive impairment.

Moreover, the density of CD4^+^ and CD8^+^ T cells was found to be increased in the dura of old mice aged 20–24 months, via CyTOF screening [26]. The spatial localization of these T cells changed from the perisinusal regions in the young mice to other dural regions in the older ones, and T cells were noted especially within the dural parenchyma [26]. Furthermore, scRNA-seq analysis revealed that the phenotypes of CD4^+^ T cells were similar between the young and aged dura, albeit with increased interferon-γ (IFN-γ) levels in the aged dural T cells [26]. These aged dural T cells were consistent with infiltration of old neurogenic niches with regard to the high expression of IFN-γ [42], regardless of the spatial orientation, suggesting a general inflammatory processes during aging. The mural or endothelial proportions were not significantly different between young and aged dura; however, the proliferative markers were expressed less in these stromal populations during aging [26]. Analysis with scRNA-seq and gene ontology (GO) analysis predicted upregulated adhesion-related pathways and disrupted extracellular matrix (ECM) in aged dural stromal populations, confirmed by the elevated induction of adhesion molecules and ECM components predominantly at non-sinus sites [26]. This process may underlie the altered spatial localization of T cells in the aged dura. Another scRNA-seq study revealed that subsets of B cells and plasma cells accumulated in the aged dura, and the former were defined as age-associated B cells (ABCs) [15]. ABCs were characterized by unique phenotypes, as shown by hundreds of DEGs between them and mature B cells [15]. Furthermore, scBCR-seq has identified an antigen-experienced pattern in dural ABCs and a minor clonal overlap shared between dural ABCs and circulating B cells, suggesting a peripheral circulation origin [15]. Dural plasma cells primarily produced IgM in aged mice, and were proven to be of non-circulating origin, and may be derived from local ABCs that undergo further differentiation [15].

### Alzheimer’s disease (AD)

AD is an age-related neurodegenerative disease clinically characterized by progressive cognitive decline. Senile plaques accumulated by amyloid-beta (Aβ) peptide and neurofibrillary tangles composed of tau proteins, are the neuropathological hallmarks of this disease [43]. Transgenic mouse models of AD expressing five human familial AD gene mutations (5 × FAD) have been widely used in this research field [44].

MLVs did not display structural changes or attenuated drainage in young 5 × FAD mice; however, meningeal Aβ deposition and macrophage increase were observed following meningeal lymphatic ablation [41]. With advancing age, the coverage of dorsal MLVs decreased, suggesting age-associated morphological impairment in 5 × FAD mice aged 13–14 months. In addition, immunofluorescence staining showed increased deposition of Aβ throughout the whole meninges [45]. Similarly, diffuse meningeal Aβ pathology and macrophage recruitment have been found in patients with AD [41], indicating that age-related meningeal lymphatic dysfunction may have a role in Aβ deposition. In addition, the meningeal lymphatic drainage function affects the outcome of Aβ immunotherapy [45], confirming its relevance in pathogenic and therapeutic mechanisms.

Furthermore, a unique microglial subtype has been recently identified in both 5 × FAD mice and postmortem brain samples from patients with AD via scRNA-seq analysis; named disease-associated microglia (DAM), this cellular type has neuroprotective potential [46]. Deleterious microglia activation presenting with DAM signature was also observed in 5 × FAD mice after meningeal lymphatic ablation [45]. In particular, DAM-conserved signatures were noted in a macrophage subtype, the number of which drastically increased during aging and in neurodegenerative conditions, as shown by integrated scRNA-seq data sets [47]. These macrophages presented highly expressed inflammation-related genes and were thus named disease-inflammatory macrophages (DIMs). DIMs were found in both 5 × FAD mice and patients with AD, especially in the leptomeninges, where abundant deposition of Aβ aggregates were also observed surrounding the local blood vessels. This finding suggests the involvement of DIMs in the meningeal inflammatory processes, consistent with their transcriptional profile [47]. Overall, the precise discrimination between protective DAM and proinflammatory DIMs, provided by scRNA-seq, yields promising cellular targets, as the latter are located in the CNS borders and are easily approachable.

## Conclusions and prospects

Currently, single-cell omics studies have provided a deeper reinterpretation of the complex components involved in meningeal immunity, elucidated their molecular mechanisms, and suggested potential functional alterations in neurological diseases.

The meninges harbor a substantial number of transcriptionally dynamic immune cells in a steady state as potential reservoirs of sentinels, releasing them in neuropathological conditions and providing the basis of meningeal immunity. These meningeal immune populations, lying at the CNS boundaries, can detect environmental signals from both the peripheral and central regions and react accordingly, exerting double-edged effects on the CNS. Innate and adaptive immune cells of different ontogenies and migration pathways may play distinct roles, even in the same neuropathological conditions. For instance, blood-derived monocytes presented with proinflammatory phenotypes, whereas adjacent bone marrow-derived monocytes demonstrated regulatory profiles in EAE, suggesting potential functional differences between distinct monocyte subpopulations [14]. Not only diverse immune cells but also stromal cells residing in the meninges collaboratively interact with each other and, therefore, enable efficient meningeal immunity. Take dural sinuses for an example: the immune hubs, where T cells recruited by stromal cell-derived chemokines keep contact with CNS-enriched antigens represented by local APCs, thus enabling immune surveillance of the CNS [26]. Accordingly, when disease-induced changes occur on one side of the meninges, the other side will also be affected, as shown by increased adhesion molecules in non-sinus stromal populations, accompanied by a skewed distribution of T cells towards non-sinus regions during aging [26]. In addition to the cellular networks, the dura-bone marrow connections and dural lymphatic vessels are crucial routes for bone marrow-derived cell migration and CSF drainage into the cLNs, respectively. Moreover, MLVs have been demonstrated to mediate the spread of neurotropic viruses from the CNS to the cLNs during viral infection [48]. Therefore, MLVs establish a direct link between the CNS and the peripheral immune system, challenging the long-held dogma that the CNS is immune-privileged. Instead, we suggest to consider the CNS as immuno-specialized. Increasing age, MLV dysfunction, turnover obstacles, and CSF protein composition changes may all be part of a vicious cycle, leading to inefficient elimination and subsequent deposition of CNS-enriched macromolecules, especially neurotoxic proteins, ultimately affecting neurological functions. These mechanisms underlie at least in part the typical pathophysiological features of several neurodegenerative diseases.

Finally, since the dura mater comprises several specific subtypes, this layer was expected to be the main contributor to meningeal immunity and CNS inflammation. However, several studies found contrary evidence; the leptomeninges appear to have a more crucial role in inflammatory processes and CNS autoimmunity [38]. Future studies accurately subdividing the individual meningeal layers should be conducted to assess each of them separately, considering their distinct structural and functional nature. The results could improve our understanding of the involvement of each meninx in brain homeostasis and diseases. The meningeal immunity protects the brain and at the same time is involved in CNS pathology, and these contrasting mechanisms should both be acknowledged.

However, several questions associated with meningeal immunity remain unanswered. For instance, the mechanisms underlying the interactions between adjacent bone marrow-derived myeloid cells and their peripheral counterparts are yet to be elucidated, as well as their involvement in various neurological diseases. Doubts remain also on the mechanisms underlying the migration of these myeloid cells within the dura to cross the remaining borders for further infiltration, considering the vascular channels without extension to the CNS parenchyma. The overall developmental trajectory of B cells in the meninges has been clarified [49]; however, whether adjacent bone marrow-derived cells undergo a similar negative selection remains to be determined, similar to the disruption of the non-self-reactive meningeal reservoir under neuropathological conditions. In addition, the possibility that the leptomeninges and CP may be indispensable to protect the CNS should be considered. The dura mater appears to have a more important role than the SDMs in other CNS perturbations, in contrast to autoimmune inflammation, and this finding merits further investigations. Future studies should also assess whether all the preliminary results on the meningeal immunity in experimental models of neurological diseases are similar in humans, and how to translate them into clinical applications. Finally, spatially resolved transcriptomics may be used to investigate spatial heterogeneity and establish a transcriptome atlas of the meningeal architecture, overcoming the inevitable loss of individual cell position information due to tissue dissociation occurring with the standard techniques [50].

Overall, single-cell techniques have remarkably deepened our understanding of meningeal immunity. The advantage of scRNA-seq compared to bulk RNA-sequencing is the ability to determine transcriptome identities at a single-cell level rather than in the average tissue depth, thus allowing the identification of transcriptional features that would be masked in bulk tissue. Nevertheless, the significant challenges arising from single-cell techniques require particular attention [51]. For example, scRNA-seq data sets typically face the problems of high sparsity, with a large fraction of zero-expressed genes. Several imputation approaches for sparse data have been implemented and evaluated [52]. Furthermore, the bath effects pose another remarkable challenge. In addition to well-designed experimental procedures, multiple batch-correction methods have been proposed and compared to determine the most suitable one [53]. Therefore, standard computational analysis methods for single-cell sequencing data sets should be further explored and optimized in the coming years.

In conclusion, our conceptual view of meningeal immunity has been reshaped thanks to new insights revealed by novel single-cell technologies. Single-cell omics will further facilitate our understanding of meningeal immunity and hopefully provide therapeutic opportunities to target its cellular and molecular mechanisms involved in neurological diseases.

## Data Availability

Not applicable.
